# LIFTOSCOPE: development of an automated AI-based module for time-effective and contactless analysis and isolation of cells in microtiter plates

**DOI:** 10.1186/s13036-023-00329-9

**Published:** 2023-02-07

**Authors:** Florian Narrog, Richard Lensing, Tobias Piotrowski, Nadine Nottrodt, Martin Wehner, Bastian Nießing, Niels König, Arnold Gillner, Robert H. Schmitt

**Affiliations:** 1 Fraunhofer Institute for Production Technology IPT, Aachen, Germany; 2 Fraunhofer Institute for Laser Technology ILT, Aachen, Germany; 3 WZL | RWTH Aachen University, Aachen, Germany

**Keywords:** Artificial intelligence (AI), Biological cells, Biomedical imaging, High throughput cell isolation, High-speed microscopy, Laboratory automation, Laser-induced forward transfer (LIFT)

## Abstract

**Background:**

The cultivation, analysis, and isolation of single cells or cell cultures are fundamental to modern biological and medical processes. The novel LIFTOSCOPE technology aims to integrate analysis and isolation into one versatile, fully automated device.

**Methods:**

LIFTOSCOPE’s three core technologies are high-speed microscopy for rapid full-surface imaging of cell culture vessels, AI-based semantic segmentation of microscope images for localization and evaluation of cells, and laser-induced forward transfer (LIFT) for contact-free isolation of cells and cell clusters. LIFT transfers cells from a standard microtiter plate (MTP) across an air gap to a receiver plate, from where they can be further cultivated. The LIFT laser is integrated into the optical path of an inverse microscope, allowing to switch quickly between microscopic observation and cell transfer.

**Results:**

Tests of the individual process steps prove the feasibility of the concept. A prototype setup shows the compatibility of the microscope stage with the LIFT laser. A specifically designed MTP adapter to hold a receiver plate has been designed and successfully used for material transfers. A suitable AI algorithm has been found for cell selection.

**Conclusion:**

LIFTOSCOPE speeds up cell cultivation and analysis with a target process time of 10 minutes, which can be achieved if the cell transfer is sped up using a more efficient path-finding algorithm. Some challenges remain, like finding a suitable cell transfer medium.

**Significance:**

The LIFTOSCOPE system can be used to extend existing cell cultivation systems and microscopes for fully automated biotechnological applications.

**Supplementary Information:**

The online version contains supplementary material available at 10.1186/s13036-023-00329-9.

## Background

Due to the growth in the market for biomedical applications, the demand for cell cultivation, observation, analysis and sorting is steadily increasing [1]. Fully automated solutions are required to keep up with the trend [2–4]. By integrating multiple automated steps into one device, synergistic effects can be exploited. To nevertheless obtain a large number of possible applications with one setup, the sub-processes should be able to be used individually [5, 6].

Developing a sophisticated fully automated, high-throughput tool for the isolation of single cells and cell cultures, and the removal of unwanted cells or particles, is still a major challenge [1, 7]. The novel LIFTOSCOPE (short for *Laser Induce Forward Transfer Microscope*, or *LIFT Microscope*) concept evolves from this conclusion. The basic idea is to assess how a device that combines cell observation, evaluation, and isolation must be designed to work efficiently, reliably, and economically. A proof of concept is shown using a prototype setup. This paper describes crucial development steps and design decisions and gives an outlook on future advancements.

LIFTOSCOPE is based on three key technologies. The setup aims for a total process time of 10 min, including each step for a complete microtiter plate, independent of the number of wells. The first key technology is high-speed microscopy with continuously moving samples for rapid image acquisition of cell cultures in standard micro titer plates (MTP) [8]. The second technology is a deep-learning-based semantic segmentation software to analyze the microscope images and localize single cells and cell clusters for isolation [9, 10].

Third, laser-induced forward transfer (LIFT) separates localized cells by transferring them contactless onto a receiver surface. This is achieved by focusing a laser beam at the interface between the microtiter plate and the cell medium below the cells. The lasers wavelength is chosen so that it is absorbed by the cell medium, which evaporises medium and creates a cavity. When this cavity collapses again, a liquid jet is formed that transfers the cells lying on top of the cavity [11].

Compared to other commercial technologies like inkjet printing, where the cell culture needs to be transferred into a cartridge for cell isolation, or laser microdissection and pressure catapulting (LMPC), which is primarily designed to transport tissue samples or fixated samples, this method allows for all the steps of oberservatoin, evaluation and isolation to be performed directly in a standard MTPs with no further sample preparation [12, 13].

This paper introduces the concept of the general setup as well as the evaluation of the key technologies. A method for a decreased transfer time with this setup by optimizing the microscope stage travel path is presented. As a proof of concept, an experiment for cell removal, a prestep to the required bottom-up transfer using LIFT has been conducted and results are presented.

## Methods

The concept describes a system that combines high-speed microscopy, image analysis, and high throughput cell isolation in one device. To address the demand of existing and future biomedical applications, the technology needs to reach both high throughput and high precision for cell analysis and isolation. Experiments have shown that high-speed microscopy can scan a complete microtiter plate in less than a minute with a resolution of 1.69 μm/Pixel. A fast and highly efficient process can be achieved in combination with LIFT, a pure optical and contact-free transfer technology, with process times in the microsecond regime for one single cell transfer.

### Hardware integration

**Fig. 1:**
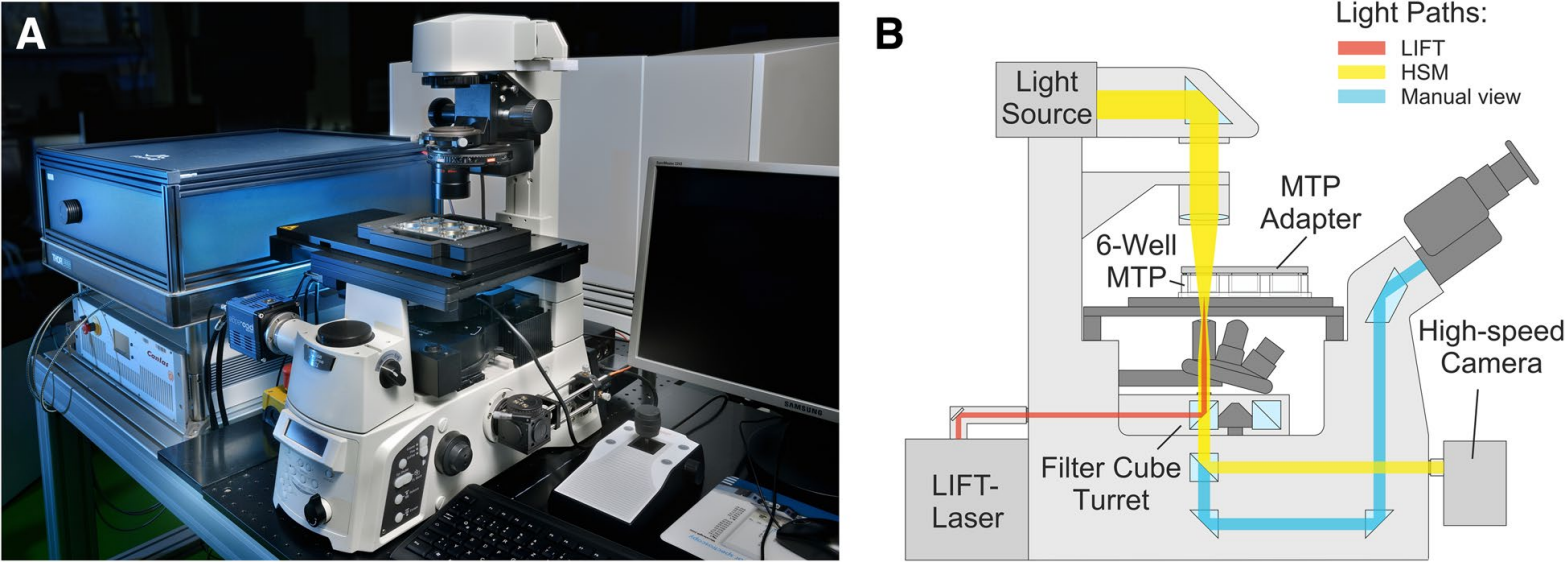
**A **Photo of the demonstrator. The demonstrator uses a Nikon Eclipse Ti 2 microscope body and a Märzhäuser Wetzlar Scan IM stage. It is controlled by the accompanying computer on the right in the picture. The laser currently used for the LIFT is a prototype system. In the final LIFTOSCOPE it can be replaced by a commercial laser source. **B **Schematics of a laser beam integrated into the light path of the inverse microscope. LIFT Laser and optical path for high-speed microscope are combined using the filter cube turret. Additionally, the standard eyepiece can be used

The integration of analysis and isolation of cells into one process is achieved by physically combining the necessary hardware in one microscopic setup. Therefore, a laser system (Nanolevante, APE Berlin, Germany) for cell isolation is added as an external unit to the microscope. The beam is led through the optical path of the inverse microscope (microscope: Eclipse Ti 2, Nikon, Japan; objective lens: CFI Plan Fluor DL 4XF, Nikon, Japan; magnification: 4, NA: 0.13) using its rear ports [14]. The concept is proven in a demonstrator (Fig. 1). By using the filter cube turret the system can switch between camera observation (camera: pco Edge 4.2, pco, Germany) and laser application. When laser mode is selected, the beam passes through the microscope’s optical path into the objective (selfbuilt objective, NA 0,2) focusing on the exact position of the cell to be transferred [15]. The moveable x,y-microscope stage (ScanPlus IM 130 × 85, Märzhäuser Wetzlar, Germany) can be used for high-speed image acquisition and cell positioning for the LIFT. The microscope can take phase-contrast images besides regular bright-field images [16]. The focal plane is adjusted by a piezo z-stage (SPS-D90500-001, nanoFaktur, Germany). All moveable components (x,y-microscope stage, piezo z-stage, filter cube turret, objective revolver) are controlled by a motion controller (TANGO 4 Dektop, Märzhäuser Wetzlar, Germany). The whole process is managed by one central computer next to the demonstrator.

### High-speed microscopy

A continuous scan process is used as proposed by Schenk [8]. Cell images are acquired during a continuous moving process. The camera allows up to 100 frames per second with a resolution of 2048 × 2048 pixels. The individual images are stitched to one image for each well, which can then be automatically analyzed.

To obtain sharp images throughout despite the microscope’s low depth of field and the uneven MTP bottom, an autofocus system is used. Contrary to the process by Schenk et al. [8], a software autofocus is implemented by acquiring a z-stack of images at multiple positions equally spread over each well [17]. At each position, the motor moves vertically to a predefined position that corresponds approximately to the focal plane. Then images are continuously taken while the piezo travels its full range. The sharpest of those images corresponds to the focal plane. Using multiple images, the surface of the well bottom can be interpolated and extrapolated to generate a focal map. During the main image acquisition process, the piezo positioning system adjusts the sample height in real time based on the previously created focal map. To further speed up the focusing process, a future implementation of the setup can include the hardware autofocus system presented by Schenk [8].

### Time-effective LIFT process

The key feature of the LIFTOSCOPE concept is the fast transfer by utilizing the continuous moving process of high-speed microscopy. The transfer process works equivalent to the image acquisition process, but instead of taking images, a laser is triggered to perform a LIFT. Therefore, the microscope stage moves along predefined paths. Synchronous to the movement of the x,y-stage, the z-stage moves according to the focal map acquired at the beginning of the microscopic scan process (see section III.B). The position of cells to be transferred is known to the algorithm as a result of the AI-based image analysis. When a target cell is positioned at the microscope’s focal point along the stage movement, a path synchronous TTL trigger signal (transistor-transistor logic) is initiated by the motion controller to start the laser for the LIFT process. The exact microscope stage position is determined by its encoder and processed by the motion controller, that also sends commands to the z-stage using an analog output signal.

Since the LIFT itself only takes around 200 μs, multiple transfers can be conducted within a second. A target of 100 cell transfers per second is set for efficient overall performance yielding a time of 100 s for an assumed 10,000 transferred cells, which is in the order of magnitude of image acquisition.

Instead of the continuous moving process described above, a ‘stop-and-go’ algorithm could more easily be implemented. Thereby, the microscope stage would move to each cell position individually and stop for every cell transfer. However, the multitude of accelerations results in the sloshing of the cell culture medium in the wells. In order to circumvent that, stop times in the order of 1 s are needed for each transfer, which drastically increases the total time.

**Fig. 2:**
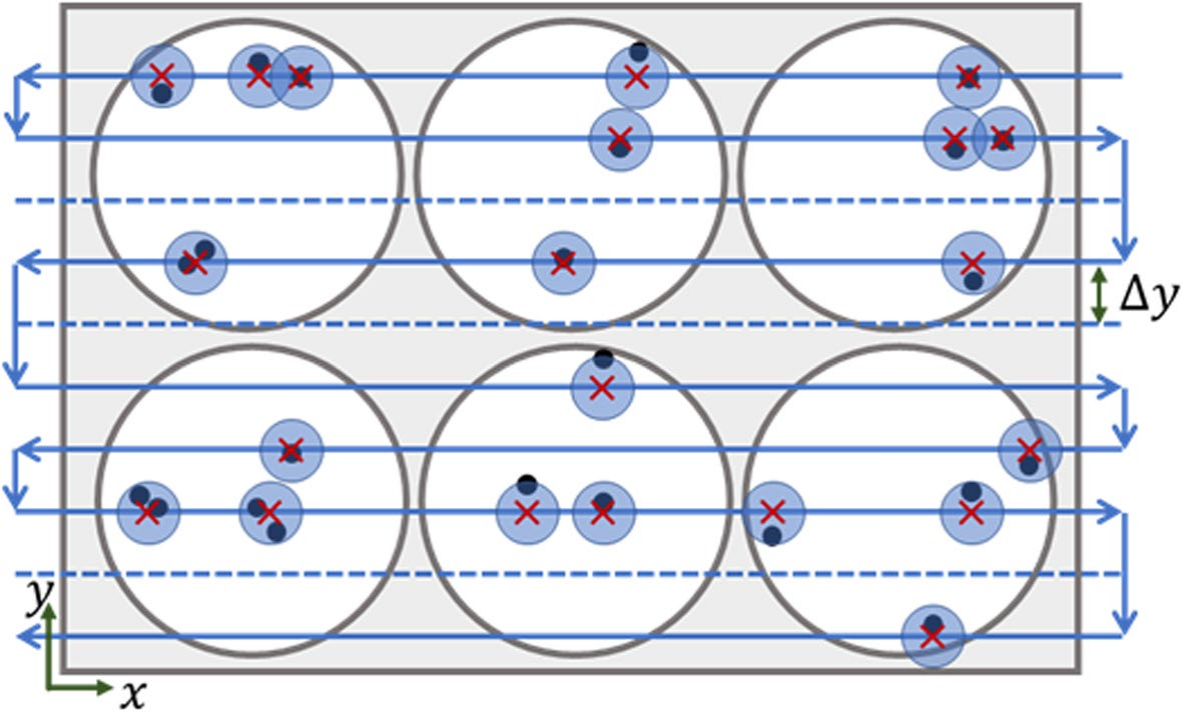
Schematic scanning path for single cell LIFT over a six-well MTP (Distance Δy not to scale). Black circles represent target cells. The laser moves along the parallel lines and is triggered if a target lies within the LIFT tolerance (blue circles around red crosses). If no target lies within the tolerance of a line, the line is skipped completely (dashed lines)

Consequently, the continuous moving method is used. To make this reliable, it has to be taken into consideration that LIFT affects an extended area around the laser focus from which cells will be transferred [18]. Assuming a radius for guaranteed cell transfer of 25 μm, this can be used to optimize the path such that not every position will precisely be reached, but only within this tolerance. Subsequently, the microscope stage will move in straight lines 50 μm apart and trigger the laser beam in an area of up to 25 μm away from the target positions. If one of the lines does not come into the tolerance area of a transferable cell, it will be skipped. This process is shown schematically in Fig. 2.

The total time for this procedure using an MTP with a usable area of 120 mm ∙ 80 mm is calculated as follows. To reduce acceleration time and achieve a higher average velocity, the number of lines must be minimized. Thus, the stage will move continuously along the long axis of the rectangular MTP. Since the area of interest is *L*_*y*_ = 80 mm wide and the spacing between two lines is Δ*L*_*y*_ = 50 μm, there are *N* = 80 mm/50 μm = 1600 lines in total. The movement time for each line can be calculated assuming constant acceleration using Newton’s equations of motion [19], which yields

1 $${\displaystyle \begin{array}{c}{t}_{line}=2 \frac{v_{max}}{a}+\frac{L_x-2 {L}_{x, accel}}{v_{max}}\\ {} for\ {L}_x\ge 2 {L}_{x, accel},\end{array}}$$

where *v*_*max*_ represents the maximum velocity and *a* the acceleration of the microscopic stage, respectively. *L*_*x*, *accel*_ stands for the distance needed to accelerate to the maximum velocity and decelerate from the maximum velocity to a standstill. It is calculated using the following formula:

2 $${L}_{x, accel}=\frac{1}{2}a{t}_{accel}^2=\frac{1}{2}a{\left(\frac{v_{max}}{a}\right)}^2=\frac{1}{2}\frac{v_{max}^2}{a}.$$

The LIFTOSCOPE demonstrator reaches *v*_*max*_ = 120 *mm*/*s* and *a* = 500mm/s^2^. To reduce sloshing, a maximum acceleration of *a* = 100mm/s^2^ is used, which thereby reduces the maximum reached velocity to *v*_*max*_ = 109.5 *mm*/*s*. These values are used for calculations, resulting in *t*_*line*_ = 2.26 s, plus the time to switch lines (*l*_*switch*_ = 0.04 s) and a pause to calm down the liquid. Conclusively, the total time is

3 $${\displaystyle \begin{array}{c}{t}_{total}=N{t}_{line}+\left(N-1\right){t}_{switch}\\ {}\approx N\left({t}_{line}+{t}_{switch}\right).\end{array}}$$

The resulting time is now compared between this algorithm and a regular stop-and-go for benchmarking. To generate enough data without screening too many real cell cultures, a Python script has been implemented to randomly distribute cells within a six-well-plate and store their positions as XML file. This file is read into two further Python scripts, calculating the transfer time with the stop-and-go and the continuous method, respectively. Studies are carried out with cell numbers of 100; 1000; 10,000; and 100,000; where five random distributions have been created each. The stop-and-go algorithm is implemented such that it works through one well after another, starting at the cell with the lowest x-value each and then moving to the coordinate with the next higher x-value. Results of this simulation are shown in section IV.

### MTP adapter

For LIFT, in addition to the MTPs containing cells to be examined, receivers are needed to which the selected cells are transferred. From there, the cells can be cultivated or examined with different methods [20, 21].

**Fig. 3:**
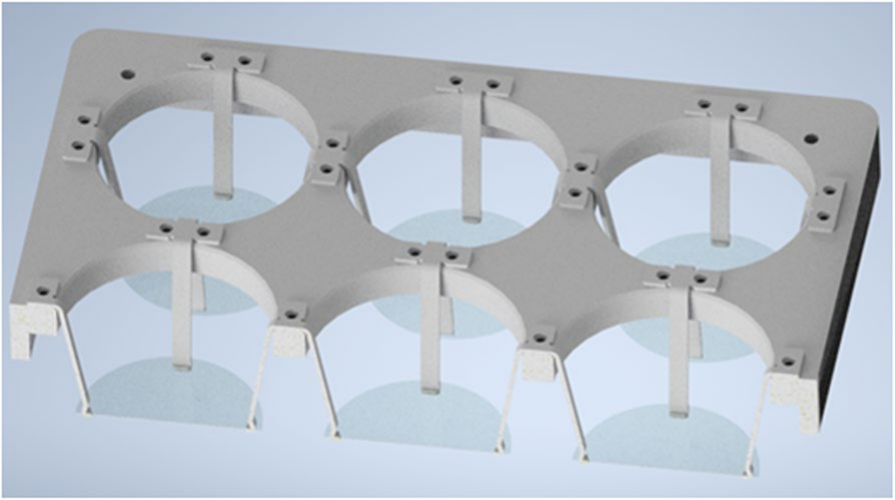
Rendering of the MTP adapter with section view. Glass plates are inserted into each well

As receivers, glass plates are chosen, positioned 2 mm above the bottom of each well. Successful high-precision transfers over this distance and greater have previously been shown [11]. Since there is no hardware to position the receiver plates for six-well MTPs, a new holder has been designed for LIFTOSCOPE experiments. It is depicted in Fig. 3 and consists of a plastic housing that fits tightly on top of a six-well MTP. Each well contains four sheet metal springs with hooks at the lower end, spaced equally around the circumference. This assembly allows the round glass plate to be mounted at a fixed distance to the well bottoms so that cells can reproducibly be transferred.

### Bottom-up LIFT with mid-infrared radiation

A pulsed laser source with pulse durations of 9 ns, pulse energies ranging from 5 μJ to 50 μJ, and a wavelength of 2940 nm is used for the LIFTOSCOPE prototype. The advantage of this wavelength is twofold. On the one hand, most polymers used for standard MTP plates show low absorption in this regime, allowing the laser to penetrate the MTP material and reach the probe. On the other hand, this wavelength matches the water absorption peak for electromagnetic radiation, hence the laser radiation is absorbed strongly in the cell culture medium surrounding the cell. This avoids the need for an additional metal or polymer absorption layer. Thus standard MTP plates can be used for the process with little preparation. The processing time of a single transfer event for an individual cell or a cell cluster in cell culture medium ranges from 10 μs to 200 μs for the transfer itself and up to 1 ms to return to the initial state [22]. This processing time increases with larger transferred volumes and distances and decreases with higher laser pulse energies [19–21]. The LIFT process is well investigated for the transfer of living cells with a cell survival rate close to 100% [23].

### Cell culture and live/dead assay

Henrietta Lacks (HeLa) epithelial cells were used to test influence of the LIFT on adherently growing cells. The cells were cultivated with Dulbecco’s modified eagle medium (DMEM) supplemented with 10% fetal bovine serum (FBS) and 100 U/mL penicillin-streptomycinat at 100% humidity, 37 °C, and 5% CO2.

The cells were stained directly after the cell removal using a solution of 8 μL fluorescein diacetate (FDA; 5 mg/mL in acetone) and 50 μL propidium iodide (PI; 2 mg/mL in Dulbecco’s phosphate-buffered saline (DPBS)) in 5 mL DPBS. After printing, the cells were rinsed with DPBS and incubated for 5 min at room temperature in the staining solution. Finally the cells were rinsed with DPBS again.

### Cell removal experiment using LIFT

In a first cell study, the influenced area for LIFT was tested in a cell removal experiment using adherent HeLa cells without a separation layer in six-well MTPs. By omitting the separation layer, no hydrogel is needed, while it is expected that the direct irradiation of the cells causes high mortality in the transferred cells.

After cultivation in a six-well MTP, the amount of cell culture medium was reduced to a thin layer of roughly 150 μm thickness to form a transfer layer. The lid of the MTP was replaced with the MTP-Adapter and cell clusters were removed using individual laser pulses. A live/dead staining of the cell culture in the MTP was conducted using fluorescein diacetate (FDA) and propidium iodide (PI). Results of the experiments are presented in section IV.

### AI-based cell analysis

Since the LIFTOSCOPE aims to analyze and separate a large variety of cells, the specific cell recognition and classification algorithms depend on the application. For this purpose, training data must be obtained and classified to train a neural network. Potentially, hyperparameters should be tuned to optimize the training results [9, 24, 25].

**Fig. 4:**
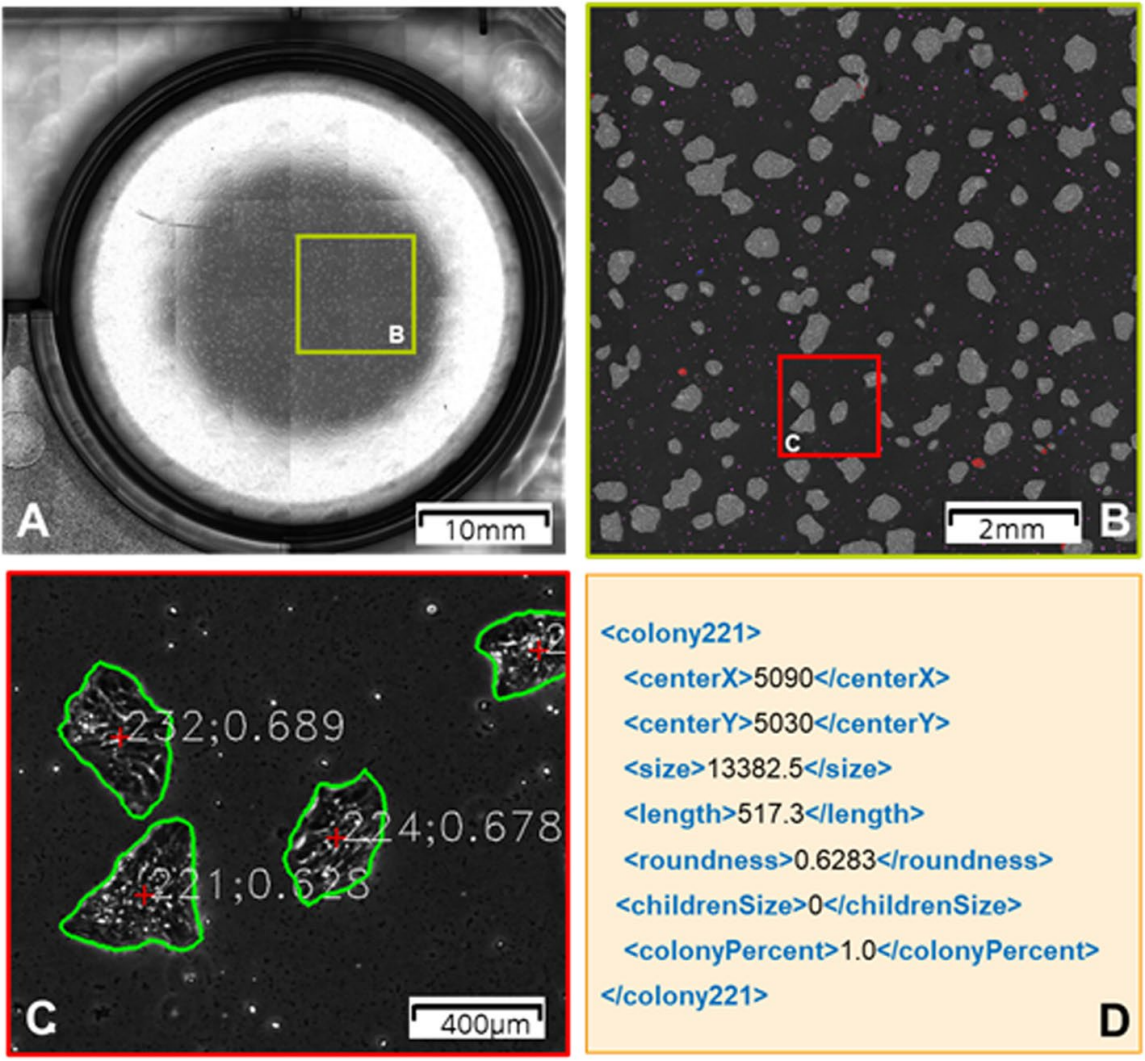
Steps of image processing for transfer preparation: **A** iPSC culture in one well of a six-well-MTP in phase-contrast microscopy with 4x objective and a resolution of 1.3 μm/px. **B** Section of the phase contrast area with multi-class segmentation via deep learning. Grey: iPSC colonies, blue: single iPSCs, black: background, purple: detached cells, red: differentiated cells, pink: dead cells. **C** Zoomed view of the colonies, border of the colonies highlighted in green, center as a red cross. The unique colony number and the roundness are written into the image. **D** XML representation using colony 221 as an example. centerX: center point X-coordinate in px, centerY: center point Y-coordinate in px, size: area of the colony in px^2^, length: circumference in px, roundness: 4π ∙ size/length, childrenSize: area of inclusions of a different class within the colony, colonyPercent: Percentage of the colony compared to the total area including inclusions

The analysis algorithm must be given images acquired by the high-speed microscope and return an array of two-dimensional target parameters for the LIFT. Thus, the algorithm must be able to select specific cells and calculate a central point, ideally the center of gravity. The neural network can either directly output a list of cells to be transferred or an XML-based property list, which is then used by a rule-based algorithm to select appropriate targets. Figure 4 shows an example of using a pre-trained algorithm to identify induced pluripotent stem cells (iPSC) [9]. It utilizes the PHANTAST algorithm to generate a foreground segmented image, which is in turn transferred into a U-Net architecture [10]. It then provides an image where all iPSCs are marked and an xml file containing data for each colony. For a proof of concept, the first task will be to identify all iPSC colonies within the region of interest and transfer them to the attached receiver plate [6].

To reduce calculation and training time of the algorithm, the image of the whole well is cropped into multiple smaller sections. The algorithm outputs local positions of cell colonies within those image sections in pixels. The algorithm for the cell transfer, however, needs absolute positions within the MTP in milimeters. Therefore, the absolute position in milimeters is calculated from the position within the image section in pixels, the section’s offset to the boundary, and the camera chip pixel size and magnification. Due to the stitching algorithm, the single frames within an image might slightly be shifted, but only in the range of less than 10 μm. The LIFT process, however, allows tolerances of up to 50 μm.

### Results and further challenges

The initial prototype setup consisting of the laser (including its periphery), the microscopic stage, and an optical path made of multiple mirrors has been assembled. It has been used to prove the general suitability of its components for a full demonstrator. Most importantly removal of cells from the MTP could be shown as described below. Therefore, this assembly is suitable for incorporating into the final LIFTOSCOPE device.

It was shown that cells can be removed reliably. Previous experiments using LIFT have shown that cells can be transferred reliably even if the laser beam is focused with a lateral deviation of 25 μm relative to the cell position [26]. This can be taken advantage of when calculating a time-optimized path for the continuously moving MTP, as presented above. However, the precision of LIFT during continuous movement must be evaluated in future tests. Moreover, studying a new algorithm which is able to transfer cells with smaller tollerances and short process times is one of the most important remaining challenges of LIFTOSCOPE.

The efficient transfer method introduced in section III.C has been compared with a stop-and-go process. The results are shown in Table 1. It can be deducted that the continuous process is more time efficient for a larger number of cells, whereas it underperformes for cell numbers up to a few thousand. This is due to the fact that the chance of multiple cells lying in a line in the direction of motion increases with the cell count. Therefore, the time to transfer 100,000 cells is only slightly higher than to transfer 10,000 cells, while this relationship is almost linear for the stop-and-go process. Moreover, a continuous process is advantages if a long pause (in the order of 1 s) is required to reduce sloshing of the liquids. For the targeted 10,000 cells and assumed 1 s pause, the continuous process is around 3.5 times faster than stop-and-go. This ratio is increased if a microscope stage with higher maximum velocity and lower acceleration is used.

**Table 1:** Comparison between stop-and-go method and continuous lift method using simulated cell positions. The average time in seconds to transfer cells of five different distributions for each number of points is shown. The influence of the pause time after each stop is examined by using two different pause times. The ratio of the resulting times (stop-and-go time divided by continuous time) is also given. The maximum velocity is assumed to be 120 mm/s, a linear acceleration of 100 mm/s^2^ is used

	Pause time = 0.1 s			Pause time = 1 s		
Number of cells	Stop-and-go [s]	Continuous [s]	Ratio	Stop-and-go [s]	Continuous [s]	Ratio
100	77.7	232.0	0.34	167.7	318.0	0.53
1000	683.3	1712	0.40	1587	2348	0.68
10,000	6799	3333	2.04	15,800	4570	3.46
100,000	67,752	3379	20.05	157,668	4635	34.03

**Fig. 5:**
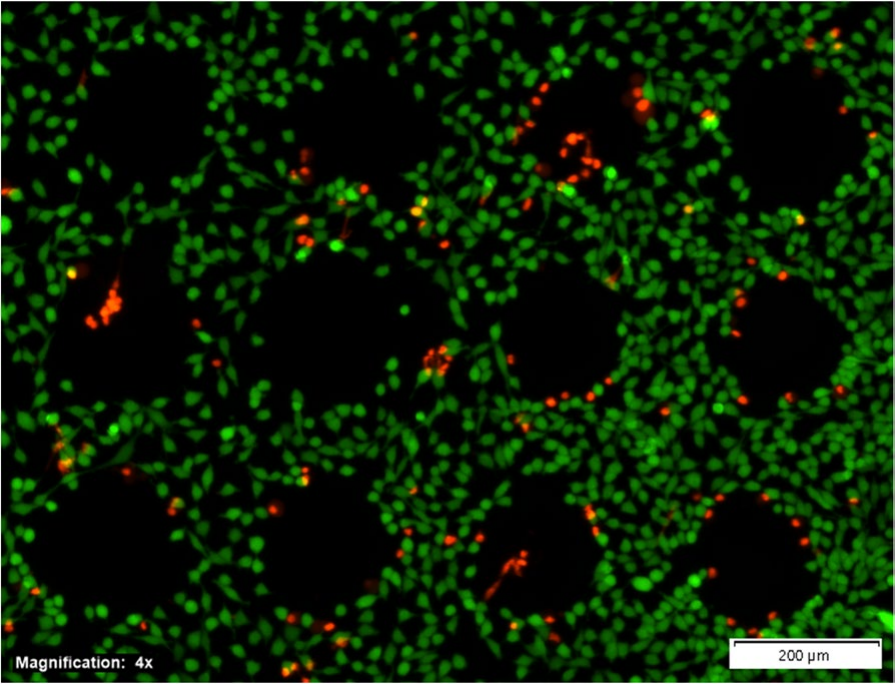
Cell culture of adherently growing HeLa cells in a six-well MTP with a live/dead staining using FDA and PI. Empty areas were induced using laser pulses with 46 μJ pulse energy and 25 μm focus diameter

Another point that has to be investigated is the transfer of adherently growing cells. To evaluate the influenced area for LIFT adherently growing HeLa cell clusters were removed from the bottom of a MTP using individual laser pulses. On the MTP, each laser pulse created an empty area of between 80 μm and 100 μm in diameter around its focus (depicted in Fig. 5). Cells outside this area were unharmed by the process, while some cells died at the edge of the area. The cells targeted for removal died during transfer, as seen in Fig. 5, first column, middle row, and third column top and bottom row. For the future, transfer of living adherent growing cells a thin hydrogel layer will be added at the bottom of the MTP plate, to avoid direct irradiation of the cells. Therefore, hydrogels and other separating layers are under investigation that are both inert and structurally stable to support the cells during cultivation and, at the same time, allow for cell adhesion. Possible candidates include, but are not limited to, Matrigel [27, 28], crosslinked gelatin [29], and chitosan [30, 31]. This separation layer will be coated on the bottom of the MTPs before the cells are cultivated and guarantees that no irradiation or thermal damage is introduced to the cells during the LIFT process. This will reduce cell damage and decrease the area of influence in the cell layer [26, 32].

The influenced area also scales with the used laser pulse energy. A larger area allows for a faster process since it gives the path-finding algorithm more freedom if a high cell transfer accuracy is not required.

Furthermore, the influence of sample movement on the LIFT process has not yet been tested. Assuming a stable velocity of 120 mm/*s* and a standard LIFT time of 200 μs/jet, the sample would move 24 μm during the LIFT process. A jet diameter of 20 μm to 50 μm will most likely result in an oblique jet. Trials to quantify this effect on the LIFT quality are currently conducted.

The AI-based cell analysis software has proven to detect iPSCs reliably and will therefore be used for the LIFTOSCOPE prototype as is [9].

## Discussion

Since LIFTOSCOPE is still under investigation, not all capabilities are known yet. There are still some challenges to overcome. The final performance of the technology cannot be quantified with certainty. However, due to the promising interim results, it can be assumed that a powerful cell sorting system is feasible.

### Technical analysis

The proposed algorithm for an optimized stage movement has been shown to be faster than a stop-and-go algorithm for 10,000 or more cells, yet the target time of 100 cells per second is still out of reach for most cell arrangements. It is assumed that due to cell clustering, a smaller share of all path lines must be traveled than in the simulations, reducing the total time, but this has to be proven by further studies. To further improve the performance of the LIFT process, more optimized path-finding algorithms will be studied and employed. The maximum tolerance for the transfer with a moving microscope stage will be determined in a preceding step since it vastly influences the path-finding. Lastly, LIFT events influence each other if they are in close proximity [18, 33], resulting in a limited maximum velocity for the microscope stage on paths where cells marked for transfer are positioned next to each other.

The image analysis software can be trained to detect various cell types. Adding new applications to the LIFTOSCOPE requires some initial effort due to neural network training, after which it can run autonomously [25]. This makes LIFTOSCOPE a versatile device whose application can be customized at the user’s site.

The most crucial objective is to ensure reliable and fast cell isolation. Cell survival under similar conditions has already been proven in multiple studies [23, 26, 32]. Since it could be shown that all individual process steps can work automatically, the goal to achieve a fully automatic overall process will likely be met.

### Applications

The LIFTOSCOPE concept presents a versatile platform that can be used in many applications. Foremost, it can be integrated into a fully automated laboratory or pharmaceutical production line [5]. LIFTOSCOPE can accelerate existing workflows due to its high throughput and complete automation. Its main advantage consists of its versatility. It can be trained to complete many cell isolation tasks, such as targeting rare cells or lifting a small number of single cells out of a large colony without fluorescence markers, which is hardly possible with other mechanisms.

Furthermore, despite being a fully integrated device, LIFTOSCOPE provides the basic functionalities of its parts. Therefore, it can still be used for manual microscopy and automated high-speed microscopy. Multiple modes are available, like transmitted light microscopy and phase-contrast microscopy. Moreover, standard MTPs can be used with little preparation needed.

While computer vision is necessary for a fully automated process, it is also possible to let the image processing algorithm only run a segmentation and then let the user decide which cells to transfer. Image analysis may also be omitted entirely, and the user conducts the whole selection process himself. This is especially needed when rare cells must be isolated, of which it is difficult or unpractical to obtain enough training data.

### Comparison with competing approaches

Even though there are approaches to separate and analyze single living cells in one device [34], no integrated high-throughput system to automatically scan, analyze and transfer is known to the authors. However, some alternatives exist to the individual process steps. The most important ones are presented in Table 2 and further discussed below. For a comparisons, pros and contras of these technologies that summarize the author’s experiences are summarized in this table.

**Table 2:** Comparison of different cell separation methods

Technology	Pro	Contra
LIFTOSCOPE	▪ Rapid imaging ▪ Overview of the entire sample ▪ High transfer precision ▪ Functions can be used individually	▪ Still in research ▪ Large investment if no microscope exists to upgrade
FACS	▪ Industry standard ▪ High throughput	▪ Cells need to be transferred into liquid sample ▪ Disturbes the cells ▪ Unsuitable for small sample volumes due to large dead volumes ▪ Fluorescence labelling necessary
Automated cell pickers	▪ High control ▪ Precise cell uptake	▪ About 20s per cell transfer
Inkjet printer	▪ Good control over cell number and deposition	▪ Cannot be used in cell cultures directly ▪ Large dead volumes ▪ Shear forces at exit nozzle negatively impact cell viability
LMPC	▪ Overview of the entire sample ▪ Transfer of large samples	▪ Can only be used with fixated samples ▪ Not suitable für single cell applications

Automated cell image analysis based on deep learning algorithms is a technology that is increasingly used due to advances in computing capabilities [35, 36]. The software deployed for the LIFTOSCOPE system is derived from a multi-class segmentation algorithm for induced pluripotent stem cells. Therefore, it can be adapted to identify most other cell types and is a perfect fit for LIFTOSCOPE [9].

There are three widely available technologies for cell sorting and isolation, aside from manual pipetting. Fluorescence-activated cell sorting (FACS) is the industry standard for cell sorting. It offers high throughput rates but needs the cells to be transferred into a liquid sample. Thus it cannot be used without disturbing the cell culture and is unsuitable for small sample volumes due to large dead volumes. Furthermore, the cells must be fluorescently labeled for this purpose. Automated cell pickers offer high control and precise cell uptake for single cells and cell clusters but require about 20 seconds per cell transfer [37]. Using an inkjet printer for cell isolation provides more control over cell number and deposition than FACS. However, it is also unable to be used in cell cultures directly and features large dead volumes. Additionally the shearforces at the exit nozzle negatively impatcs the cell viability [38]. Lastly laser microdissection and pressure catapulting (LMPC) needs to be mentioned since the setup with a laser system integrated into a microscope looks similar to LIFTOSCOPE. However LMPC is not designed to be used with single cells and cell cultures in liquid medium and needs fixated cells or a other semi-solid media for the transfer.

In cases where a small to medium cell number needs to be isolated from a large pool of cells, the LIFTOSCOPE technology can exceed existing technologies by providing a high-throughput rate with low dead volumes and high transfer precision directly from the cell culture plate.

## Conclusion and outlook

This paper presents a concept for fully automated cell analysis and isolation. Due to its modular design, it is highly versatile, and existing microscopes can be updated with its functionalities. By integrating the existing high-speed microscopy, AI-based image recognition, and LIFT technologies, fast image acquisition, analysis, and gentle cell transfers can be ensured. The concept can be applied to a large variety of applications for different cell types, and many regular microscopes can be upgraded to work with the LIFTOSCOPE principle. Further experiments will be conducted to study LIFTOSCOPE under real conditions.

## Supplementary Information

**Additional file 1.**

## Data Availability

Because LIFTOSCOPE is a publicly funded research project, all relevant findings are publicly available. Functionalities are described so that they are comprehensible for the reader. The high-speed microscopy control source code, however, is intellectual property of Fraunhofer IPT. A Fraunhofer ILT patent for the use of mid-infrared laser sources in laser assisted bioprinting is currently pending.
